# Post-operative Patellar Tilt More than 10° Can Affect Certain Components of Knee Society Score After Total Knee Arthroplasty at 2-Year Follow-Up

**DOI:** 10.1007/s43465-023-01077-0

**Published:** 2024-02-19

**Authors:** Swapnil Singh, Toh Mingzhou, Jichuan Wang, Lingaraj Krishna

**Affiliations:** 1 Department of Orthopedics Surgery, University of Alabama, 1812 4th Ave, S #308, Birmingham, AL 32533 USA; 2 Division of Sports Medicine and Surgery, Department of Orthopedic Surgery, Hospital Sports Centre, National University Health System, NUHS Tower Block, Level 11, 1E Kent Ridge Road, Singapore, 119288 Singapore; 3 Musculoskleletal Tumor Center, Beijing Key Laboratory for Musculoskeletal Tumors, Peking University People’s Hospital, Beijing, China

**Keywords:** Patella tilt, Total knee arthroplasty, Functional outcome

## Abstract

**Introduction:**

The effect of post-operative patella tilt on functional outcomes after total knee arthroplasty remains unclear. Our study aimed to analyze the relationship of post-operative patellar tilt with functional outcome scores after total knee arthroplasty.

**Materials and Methods:**

Patient data were retrieved from our institution’s prospectively maintained total knee arthroplasty. Three hundred three patients who underwent unilateral TKA from Jan 2012 to March 2017 were included in the study. After excluding patients with incomplete and lost follow-up data, 213 patients were analyzed. Radiographs of pre-operative and post-operative skyline views were used for patella tilt and patella displacement measurement at pre-op, post-op 1 year, and post-op 2 years. Three functional outcome scoring systems, SF-36, KSS, and WOMAC, were applied for function evaluation at different post-operative time points. Patients were divided into three subgroups according to the patella tilt, which includes less than 5°, 5.1–10°, and more than 10°. Statistical analysis was done to identify the relationship between patella tilt and functional outcomes.

**Results:**

Mean post-operative patella tilt was significantly lower than the mean pre-operative patella tilt (3.35 ± 3.91 vs. 5.65 ± 4.41, *p* < 0.001). There was no significant difference in patella displacement among pre- and post-operative status. KSS functional score was significantly poor at post-op 1 year and KSS objective score at post-op 2 years in patients with more than 10° patella tilt. SF-36 and WOMAC were not significantly different among the groups. There was no significant difference in post-operative function between non-resurfaced and resurfaced patella patients evaluated with three scoring systems.

**Conclusion:**

We have found significantly less post-operative patella tilt after TKA than pre-operative patella tilt with or without patella resurfacing. Increased post-operative patella tilt of more than 10° can affect specific functional outcomes. Patella resurfacing does not affect the post-operative functional outcome compared to non-resurfacing of the patella post-op 2 years.

**Level of Evidence:**

III.

**Supplementary Information:**

The online version contains supplementary material available at 10.1007/s43465-023-01077-0.

## Introduction

Total knee arthroplasty (TKA) is one of the most common elective orthopedics procedures worldwide [1]. With the recent advances in surgical techniques, implant design, and instrumentations, arthroplasty surgeons aimed to achieve anatomical and biomechanical restoration of prosthetic knee joints intra-operatively and the best functional outcome post-operatively. However, studies suggested mixed results in patient satisfaction after surgery [2, 3]. Possible reasons include unmet expectations, functional limitations, and post-operative complications such as pain and swelling [4, 5]. Post-operative knee pain was identified as a cause of dissatisfaction in both resurfaced and non-resurfaced patella patients and was considered unrelated to the patella cartilage condition [6, 7]. Common causes of anterior knee pain were identified as patellofemoral complications, including patellofemoral maltracking, aseptic loosening, fracture of the patella, and avascular necrosis [8–11]. These complications are the second most common reason for revision surgery after TKA [12–14]. Few studies have mentioned the effect of the implant design and their position and alignment on anterior knee pain because of patellofemoral complications [14–17]. Recent advances with improved design of the trochlear groove and techniques to avoid malposition and malrotation are well documented in the literature [18–21]. However, there is paucity and controversies on evidence regarding patella tilt and maltracking on functional outcomes after TKA.

Traditionally, patient-reported outcome scores are used to evaluate post-operative function after TKA by comparing pre- and post-operative scores [2, 9, 22, 23]. Previous studies also favor using these scoring systems to objectively and subjectively assess patient satisfaction [2, 24]. The post-operative skyline view facilitates the identification of patellofemoral tracking, including patellar tilt (PT), patellar displacement, patella resection angle, and combined resection angle of the patella [15, 17]. It raises concern among surgeons whether these parameters affect post-operative functional outcomes and if there is any evidence on the upper limit of these parameters related to post-operative poor functional outcomes.

Therefore, the primary aim of this study was to identify the relationship between post-operative functional outcomes with patella tilt and patella displacement after total knee arthroplasty.

## Materials and Methods

Three hundred three patients in the study underwent primary total knee arthroplasty surgery from Jan 2012 to Mar 2017. Seventy patients were excluded because of incomplete data on functional scores, fifteen patients were excluded because of the unavailability of necessary radiographs, and five patients were excluded as deceased at the time of analysis. Two hundred thirteen cases were included in the outcome analysis 2 years after the surgery. All the patients had consented to participating in retrospective research and using data from imaging and patients-reported outcomes before surgery. Demographic variables were listed but not included in the comparison (Table 1). Operative information was collected from medical records in the hospital’s computerized records system. Patient-reported outcomes were used pre-operatively and post-operatively as described [25]. These include Knee Society Score (KSS), Short Form-36 (SF-36), and Western Ontario McMaster University Osteoarthritis Index (WOMAC). The study was approved by the institutional review board (IRB) and the national domain-specific review board (DSRB) of the National University Hospital System (NUHS) under the reference ID number 2019/00877.

**Table 1:** Demographic and clinical evaluation of the cohort

Knees (*n* = 213)	Mean ± SD
Age (years)	60.40 (range 51–85)
Gender	
Female	168 (79%)
Male	45 (21%)
Pre-operative mechanical alignment	Not enough data available
Post-operative mechanical alignment (*n*1 = 189)	
− 3° to 3° (*n* = 130)	
< − 3° to − 10° (*n* = 18)	
< − 10° (*n* = 2)	
> 3° to 10° (*n* = 39)	
> 10° (*n* = 0)	
Resurfaced patella	104 (49%)
Non-resurfaced or native patella	109 (51%)
Follow-up	4.2 years (range 2–6.3 years)

*n*1 number of patients with post-operative lower limb scanogram

All patients were operated on by the senior author (LK) with the cemented posterior-stabilized implant (NRG Scorpio, Stryker, USA). All TKAs were performed via a standard anterior midline incision and medial parapatellar approach. Bony landmarks, including TEA (trans-epicondylar axis) and AP (anteroposterior), were utilized to set the femoral rotation component using the measured resection technique. Mechanical alignment in the coronal plane was established using an intramedullary alignment guide for both the femur and tibia sequentially. An anterior reference system was used to determine the size of the femoral component. The tibial component rotation was established using an intramedullary alignment guide matching the anterior part of the medial tibial plate with the anteromedial contour of the tibial cut and the center of the tibial plate aligned with the center to medial third of the tibial tuberosity. Tibial component alignment was rechecked with an extra-medullary rod attached to the tibial baseplate.

The decision for patella replacement was taken intra-operatively according to the cartilage status. The patella was not resurfaced without cartilage defects or small AP width (< 20 mm). Circumferentially denervation of the patella using electrocautery was done in routine after everting. After removing osteophytes, the patella was prepared using a patella cutting guide. After placing all joint components, patella tracking was assessed using the “no thumb technique” and the “towel clip test” during the entire range of motion. When patella tracking was found inadequate, a partial lateral patella facetectomy was performed to improve patella tracking. We did not find the necessity of lateral retinacular release to improve patella tracking in this cohort. After satisfactory tracking, the arthrotomy was closed in extension with continuous running and intermittent locking with suture. All patients received local infiltration of a cocktail of analgesics without steroids depending on drug allergy status [26]. All patients received similar post-operative pain control management and hospitalization for 3–4 days.

All patients received pre- and post-operative skyline views at 30°. Radiographic measurements were made using tools on the digital imaging software for pre- and post-operative assessment, including patellar tilt (pre- and post-op), patellar displacement (pre and post-op), resection angle (post-op), and combined patella tilt (post-op) in skyline views. Patella tilt and patella displacement on the skyline view are reproducible measurements of the patellar position on a skyline axial radiograph following a well-functioning TKA [27]. All the data were documented for further statistical analysis.

**Fig. 1:**
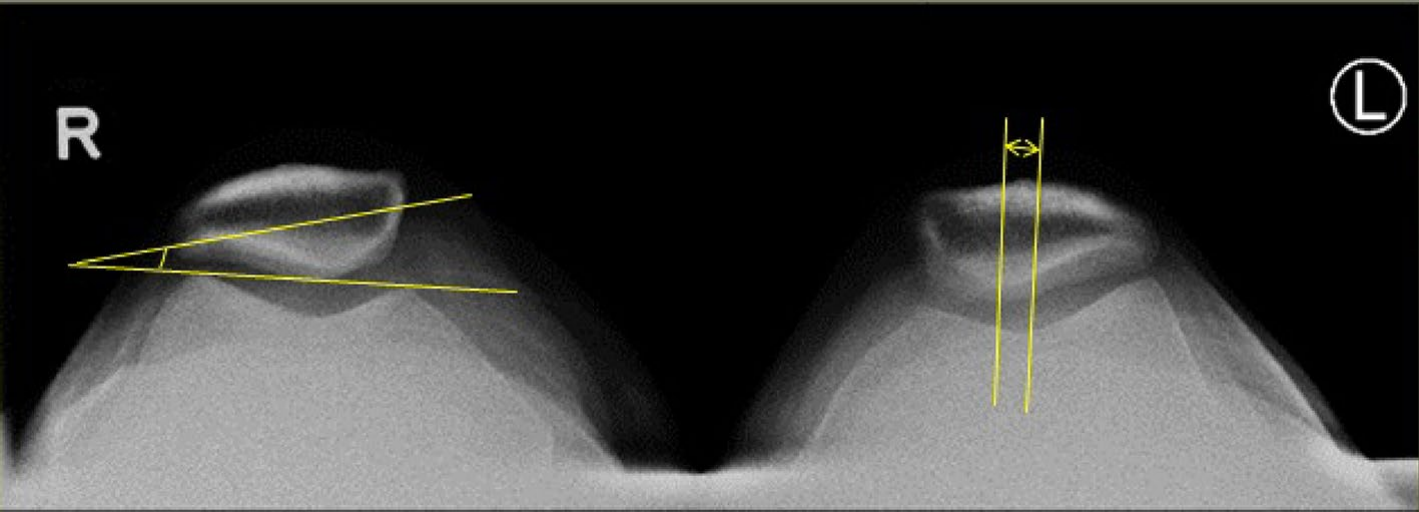
Pre-operative patella tilt in the right knee and patella displacement in the left knee

**Fig. 2:**
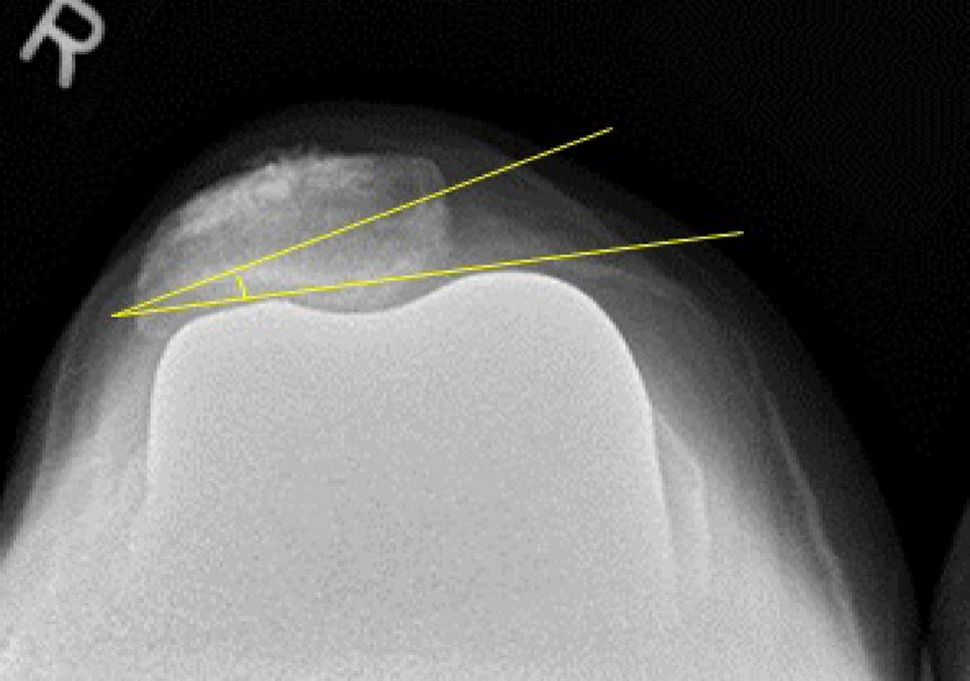
Post-operative patella tilt in TKA with non-resurfaced patella

**Fig. 3:**
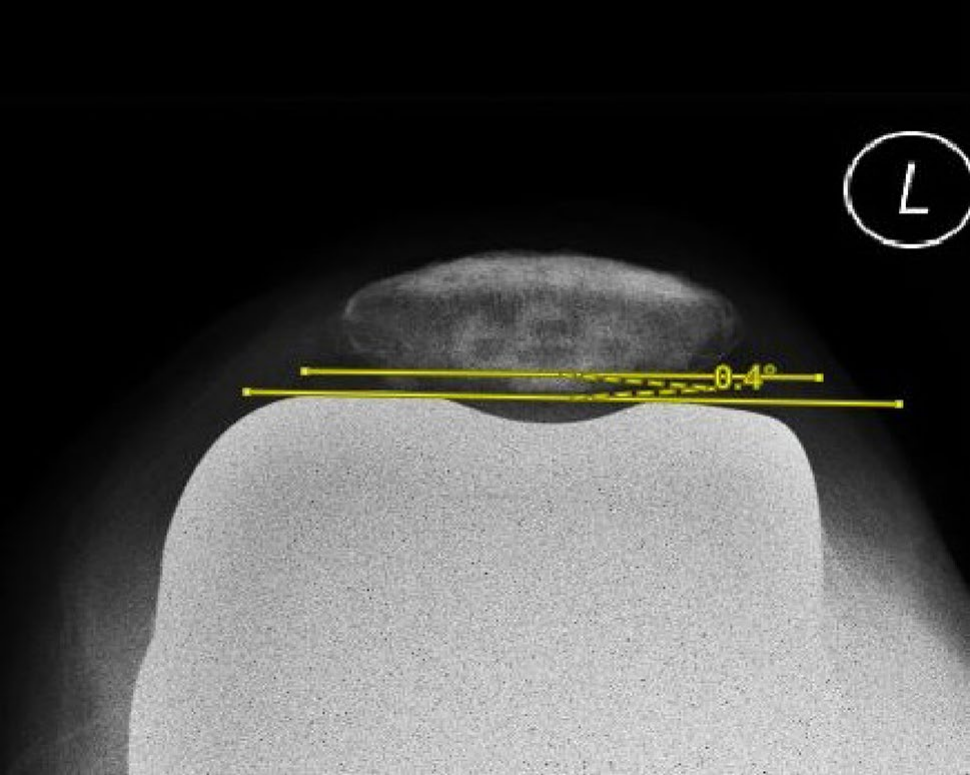
Post-operative patella tilt in TKA with resurfaced patella

**Fig. 4:**
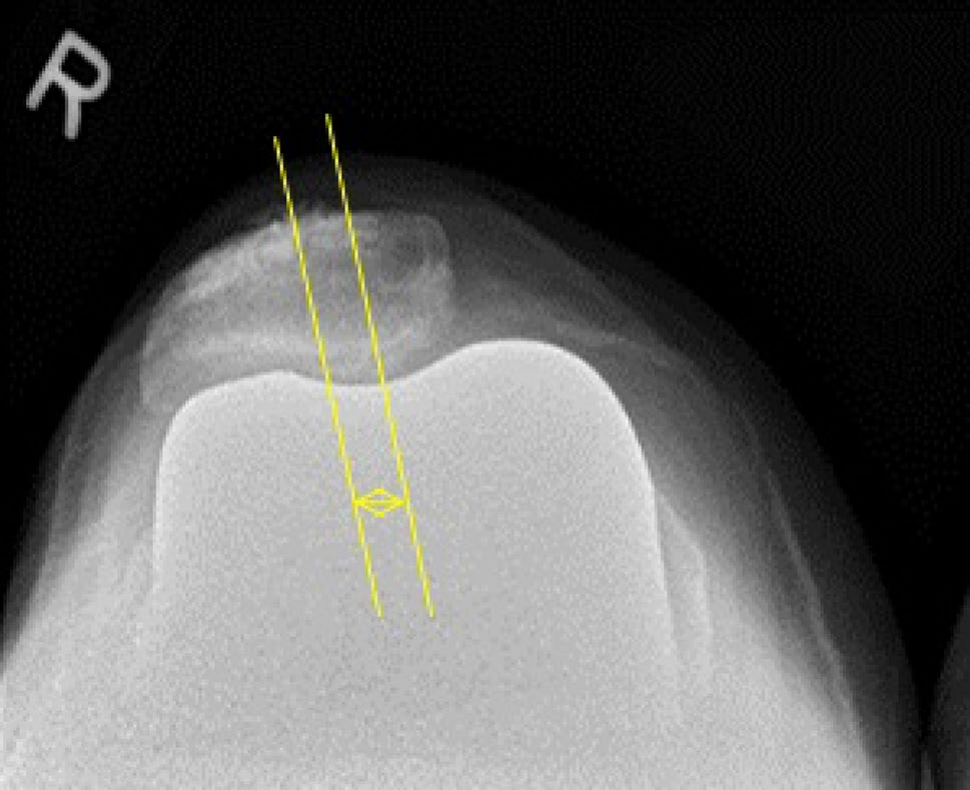
Patella displacement after TKA

**Fig. 5:**
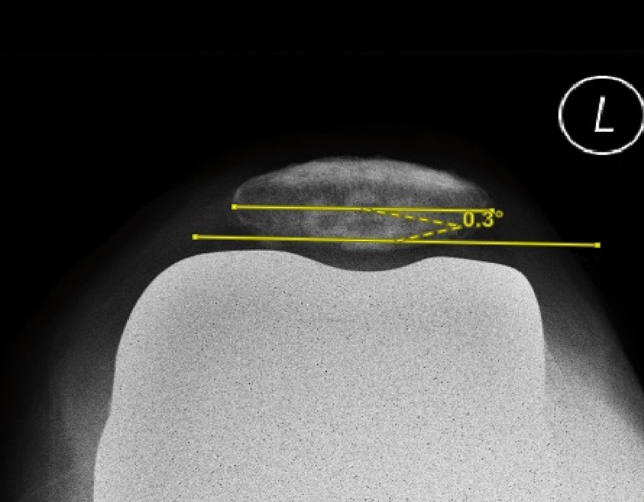
Resection angle in post-operative TKA

**Fig. 6:**
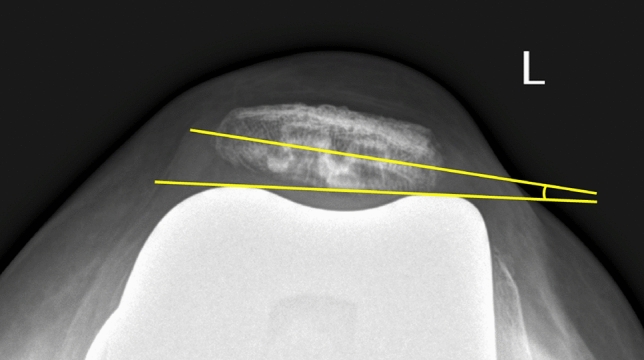
Combined patella tilt after TKA in non-surfaced patella group

In this study, the pre-operative patellar tilt was defined as the angle between a line drawn from the anterior limits of the femoral condyles and a line drawn from the posterior limits of the articular surfaces of the medial and lateral facets of the patella (Fig. 1). Post-operative patellar tilt was defined as the angle between a line from the anterior limits of the femoral condyles and a line drawn down through the prosthesisbone interface of the patella in resurfaced patella group or along the long axis of the patella in the non-resurfaced group. (Fig. 2, 3). Patellar displacement was measured as the distance between two parallel lines perpendicular to the femoral component, one through the patellar apex and the other through the trochlear apex (Fig. 4). Lateral displacement was considered positive. The patellar resection angle was defined as a line along with the bone–prosthesis interface through the middle of the patella remnant (Fig. 5). A combined patellar tilt (CPT) was defined as the sum of the patellar resection angle and patellar component tilt (Fig. 6) [20, 28].

### Statistical Analysis

Categorical variables are presented as mean and standard deviation (SD) and were assessed using the Chi-squared test, while categorical variables are presented as numbers and percentages and were assessed via Student’s *t* test. Descriptive statistics are displayed as means with standard deviations for continuous variables and frequencies with percentages for categorical variables. Differences between pre- and post-operative patellar tilt, patellar displacement, and clinical outcome scores were assessed using Student’s *t* test. One-way ANOVA was used to compare the statistical difference between groups for the above variables. The post hoc comparison of pairwise means was made using Tukey’s range test. The post hoc sample size analysis was done assuming two-tailed testing. A sample size of 41 participants was needed per group to reach an alpha level of 0.05 and a power of 80%. The level of significance was set at *p* < 0.05. STATA Version 15.0 (STATA, StataCorp, USA) was used for statistical analysis.

## Result

Two hundred thirteen patients were included finally. All patients were between 51 and 85 years of age, with a mean age of 60.40 years (Table 1). Among all patients, there were 45 males (21.0%) and 168 female (79.0%) patients. One hundred four patients’ patella resurfaced, while one hundred nine patellae were left intact. Thirty-one patients required partial lateral facetectomy. There was a significant improvement in post-operative patella tilt compared to pre-operative patella tilt (3.35 ± 3.91 vs. 5.65 ± 4.41, *p* < 0.001) (Table 2). There was no significant difference in patella displacement in the post-operative period compared to the pre-operative period (*p* = 0.13) (Table 2). We did not have enough radiological data to assess pre-operative mechanical alignment. Post-operative standing bilateral lower limb scanograms were available in 189 patients. One hundred thirty patients had neutral alignment from − 3.0° to 3.0°. Thirty-nine patients had a valgus alignment of more than 3°, while twenty patients had a varus alignment of more than − 3.0°. There was a significant improvement in all post-operative functional scores compared to the pre-operative score (Table 3). We have further divided patients into three subgroups according to post-operative patella tilt as less than 5°, between 5° and 10°, and more than 10°. In further multivariate analysis, we found that there was no significant difference in functional outcome scoring systems except KSS functional score, which was significantly worse at post-operative 1 year in PT greater than 10° (*p* < 0.05), and KSS objective score, which was considerably worse at 2 years (*p* < 0.05) (Table 4).

**Table 2:** Comparison of radiological parameters in the pre- and post-operative period

	Pre-op (mean ± SD)	Post-op (mean ± SD)	Mean diff ± SD, *p* value
PT (*n* = 213 knees)	5.65 ± 4.41	3.35 ± 3.91	2.29 ± 0.29, < 0.001
PD	0.39 ± 3.12	0.08 ± 0.25	0.31 ± 3.12, 0.13
PT + CPT^a^	5.65 ± 4.41	3.93 ± 4.08	1.71 ± 0.38, < 0.001

*PT* patellar tilt, *PD* patellar displacement, *CPT* combined patellar tilt, *SD* standard deviation

^a^PT of 109 knees and CPT of 104 knees

**Table 3:** Comparison of post-operative functional scores with pre-operative functional scores

	Pre-op (mean ± SD)	Post-op 1 year (mean ± SD)	Mean Diff ± SE, *p* value	Post-op 2 year (mean ± SD)	Mean diff ± SE, *p* value
PCS	31.77 ± 6.98	47.66 ± 7.18	− 15.89 ± .56, < 0.001	48.045 ± .48,	− 16.28 ± .66, < 0.001
MCS	56.79 ± 7.52	58.39 ± 4.42	− 1.54 ± .57, 0.007	58.99 ± 3.85	− 2.14 ± .58, < 0.001
KSS− Knee	37.83 ± 15.53	92.33 ± 12.67	− 54.50 ± 1.33, < 0.001	93.50 ± 10.49	− 55.66 ± 1.35, < 0.001
KSS-function	50.16 ± 18.86	75.52 ± 17.64	− 25.35 ± 1.70, < 0.001	76.93 ± 16.75	− 26.77 ± 1.69, < 0.001
WOMAC	62.55 ± 12.61	88.36 ± 8.90	− 25.80 ± 1.01, < 0.001	89.07 ± 6.88	− 26.51 ± .97, < 0.001

*PCS* Physical Component Score of SF-36, *MCS* Mental Component Score of SF-36, KSS-knee is the part 1 of KSS, KSS-function is part 2 of KSS, *SF-36* Short Form-36, *KSS* Knee Society Score, *WOMAC* Western Ontario McMaster University Osteoarthritis Index

**Table 4:** Comparison of post-operative functional scores within three divided subgroups according to patellar tilt degrees

With PT of 213 knees	PT ≤ 5° (mean ± SD)	PT 5.1°–10° (mean ± SD)	PT > 10° (mean ± SD)	*p* value (PT > 10°)
PCS 1	48.04 ± 7.09	46.58 ± 7.70	47.77 ± 5.84	0.421
MCS 1	58.65 ± 4.20	57.78 ± 4.93	57.93 ± 3.83	0.414
KSS-knee 1	91.96 ± 14.22	93.54 ± 7.82	91.57 ± 10.41	0.714
KSS-function 1	77.45 ± 16.68	72.10 ± 19.03	67.50 ± 19.38	**0.031**
WOMAC 1	88.95 ± 7.58	86.85 ± 11.81	87.86 ± 9.01	0.307
PCS 2	48.33 ± 6.91	47.25 ± 7.41	48.19 ± 8.14	0.627
MCS 2	59.26 ± 3.13	58.57 ± 5.33	57.97 ± 3.55	0.311
KSS-knee 2	93.29 ± 10.07	95.93 ± 6.30	85.83 ± 20.81	**0.010**
KSS-function 2	78.64 ± 14.29	73.03 ± 21.37	75.00 ± 17.75	0.094
WOMAC 2	89.30 ± 6.73	88.71 ± 7.07	88.22 ± 7.94	0.775

With PT of 109 knees and CPT of 104 knees	PT ≤ 5° (mean ± SD)	PT 5.1°–10° (mean ± SD)	PT > 10° (mean ± SD)	*p* value (PT > 10°)
PCS 1	48.00 ± 7.25	46.92 ± 7.35	47.69 ± 5.90	0.595
MCS 1	58.54 ± 4.37	58.14 ± 4.73	58.07 ± 3.63	0.800
KSS-knee 1	92.78 ± 12.18	91.81 ± 14.08	90.5 ± 11.34	0.735
KSS-function 1	79.31 ± 14.09	73.93 ± 18.02	68.75 ± 18.57	**0.012**
WOMAC 1	88.96 ± 7.68	87.31 ± 11.14	87.27 ± 8.88	0.400
PCS 2	48.43 ± 6.87	47.31 ± 7.44	47.84 ± 7.79	0.576
MCS 2	59.18 ± 3.08	58.94 ± 5.03	57.66 ± 4.02	0.328
KSS-knee 2	94.14 ± 8.05	94.32 ± 10.55	85.28 ± 20.31	**0.009**
KSS-function 2	78.63 ± 14.40	73.71 ± 20.36	76.25 ± 16.98	0.147
WOMAC 2	89.70 ± 6.00	88.05 ± 8.16	88.18 ± 7.47	0.247

All scores at post-operative 1 year and 2 year are named by suffix, e.g., PCS 1 is the PCS score at post-operative 1-year follow-up, PCS 2 is the PCS score at post-operative 2-year follow-up

Significant differences (*p* < 0.05) are in bold

*PCS* Physical Component Score of Short Form-36, *MCS* Mental Component Score of Short Form-36, *KSS-Knee* the part one of Knee Society Score, *KSS-Function* the part two of Knee Society Score, *WOMAC* Western Ontario McMaster University Osteoarthritis Index, *PT* patellar tilt, *CPT* combined patellar tilt

We did further analysis taking combined patella tilt (CPT) into account for patients who receive patella resurfacing as the measurement of resection angle and provides better information regarding the position of the patella for femoral condyle with PT of the rest of the patients. We again found significant improvement in post-operative patella tilt (3.93 ± 4.08 vs. 5.65 ± 4.41, *p* < 0.001) (Table 2). We divided patients into three subgroups considering CPT as a measure of actual patella tilt in resurfaced patients, along with PT in the rest of the patients. There was no significant difference in functional outcome among all three groups, except KSS functional score at post-operative 1 year and KSS objective score, which was significantly worse at post-operative 2 years in PT greater than 10° (*p* < 0.05, Table 4).

We did not find significant differences in functional outcomes between un-resurfaced and resurfaced patella at post-operative 1 and 2 years (Supplemental Table 2). Neither did we find significant differences in post-operative PT and CPT in both groups (Supplementary Table 1). There was no post-operative patella fracture or dislocation discovered in the follow-up.

## Discussion

The causes and consequences of patella maltracking on anterior knee pain were partly reported previously. However, limited studies provide evidence on post-operative patient satisfaction and its relationship with patella tilt. There are multiple causes for the lateral patella tilt, including internal rotation of the femoral component, external rotation of the tibia component, valgus malalignment, and soft tissue imbalance. Combined femorotibial internal rotation was reported to be correlated with lateral patella tilt, as 1°–4° of rotation correlated with greater patella tilt, 3°–8° associated with patella displacement (greater than 0°), and 7°–18° coupled with patella dislocation or late prosthesis failure [29]. We aimed to determine the effect of patella tilt on post-operative patient satisfaction by continuously using three independent patient-reported outcome scoring systems up to 2 years after the surgery. The most important finding from the present study is that there was a significantly poor KSS functional score at 1 year and KSS objective score at 2 years post-operatively associated with a patella tilt of more than 10°. On the other hand, there was no significant difference found in the other two functional scoring systems, including the SF-36 (both physical and functional components) and the WOMAC scores in 2 years post-operatively.

These findings were contradictory to a previous study done by Narkbunnam et al. In a retrospective review of 138 primary TKA cases with patellar resurfacing, they found the odds ratio of a poor outcome score with suboptimal patellofemoral mechanics as 3.4 (95% CI 1.6–7.2) for KSS, 6.4 (95% CI 2.9–14.2) for the Knee injury and Osteoarthritis Outcome Score (KOOS), and 5.9 (95% CI 2.6–13.5) for WOMAC [30]. Suboptimal patellar tilt was defined as a patellar tilt of more than 5° and lateral patellar displacement of more than 5 mm [31, 32]. They supported the results from CT-based study done by Bells et al. in which they identified internal rotation malalignment of tibial (*p* = 0.0003) and femoral (*p* = 0.014) components individually as well as combined component rotation (*p* = 0.0003) and component rotation mismatch (*p* = 0.0001) to be a factor in pain following TKA [33]. The above results were also supported by findings from another study by Matsuda et al. [34]. However, in a retrospective study by Young et al. to evaluate unexplained knee pain following TKA, there was no difference in the incidence of tibial or femoral component malalignment in painful vs. well-functioning TKAs [35]. Later this finding was supported by Becker et al., who reported that internal and external malrotation of the femoral component does not correlate automatically with poor knee function [36]. However, in the same study, patients with additional internally rotated femoral components scored worse in the physical function category of WOMAC at 6 and 24 months post-operatively. A systemic review by Corona et al. recently showed that malrotation of the femoral component does not correlate with poor functional outcomes automatically [37]. In contrast, another systemic review by Shiavone Panni et al. showed that internal rotation of the tibia by more than 10° might be a significant factor for pain and inferior functional outcome [37, 38].

Patella tilt and patella displacement were also studied previously in relation to patella thickness and facet angle, as well as their effect on post-operative functional outcome and osteonecrosis. A pre-operative patella facet angle of less than 126° was correlated to increased post-operative patella tilt compared to greater than 126° by Inoue et al. [39]. The former group was found to have more frequent development of progressive osteosclerosis of the patellar ridge at 5-year follow-ups associated with pain and functional impairment. Compared to this study in cruciate-retaining total knee arthroplasty, Kim et al. did a similar survey in posterior-stabilized total knee arthroplasty. They found that patella shape evaluated by patellar facet angle can partially affect the pre-operative patellofemoral alignment. They indicated the insignificant clinical relevance of the patella shape in posterior-stabilized total knee arthroplasty. Hence, radiologic and clinical outcomes evaluated after posterior-stabilized total knee arthroplasty showed little difference among various patella shapes [40].

We used the 30° axial view to assess the patellofemoral congruence via the skyline technique. This technique is well adopted in pre- and post-operative evaluation protocols in the majority of institutions around the world. A retrospective study of 90 patients following primary total knee arthroplasty by White et al. used end-on-axial view to calculate patella tilt, lateral patella displacement, and patella overstuffing. They evaluated the relationship between radiographic risk factors for the anterior knee with an anatomic patella button. An increased combined patella tilt was found as a risk factor for developing anterior knee pain and painless noise in a follow-up of more than 2 years. However, they failed to identify patellar tilt, displacement, or overstuffing as risk factors for adverse clinical outcomes [21]. They did not find a correlation between patella tilt and post-operative functional outcome after patella resurfacing supporting results from the previous studies with different designs of the patella button [15, 41]. Nonetheless, they found greater patella resection angles only statistically significant as an independent risk factor for both anterior knee pain and painless noise.

Presently, there are still controversial shreds of evidence regarding the effect of patella tracking on functional outcomes. To our knowledge, this is the most extensive study that compared radiological parameters of patella tracking with functional outcomes using three independent patient-report scoring systems. The strength of the study includes a large sample size with heterogeneous case distribution. Hence, analytical data were drawn from prospectively maintained institutional registry databases, reducing the risk of observer bias. In addition, the surgeon, data observer of the registry, and statistician were all blinded to patients’ information and follow-up results.

There were also several limitations and biases in the study. First, the study framework was a retrospective analysis of consecutively operated patients from a single institute. However, the result was strictly based on data from the registry at 1 and 2 years of follow-up and was not artificially correlated with clinical signs, including pain and range of motion. The study aimed only to identify the relationship between functional outcomes and post-operative patella tilt. Second, the knee CT scan may be a better modality to identify component malposition and analyze reasons for patella maltracking. However, we used a 30° skyline view to identify patella tilt and its correlation with functional outcomes, avoiding additional radiation exposure to the patients. Third, since only one type of implant was used in this study, selection bias should be considered when interpreting the results. Fourth, we did not have enough data to compare pre- and post-operative mechanical alignment to interpret its effect on patella tilt. Fifth, we found PT and CPT are a close representations of patella tilt in two different groups but are not identical. Sixth, we did not assess effect of patellar thickness and stuffing on functional scores as they were included in original protocols of the study. Lastly, we have not identified the relationship between other factors, including age, gender, BMI, symptoms, and patella shape and stuffing, with post-operative functional outcome and patella tilt. The study aimed to identify whether patella tilt and displacement affect functional outcomes. Although our research suggests patella tilt greater than 10° is an adverse prognostic factor for post-operative function by the KSS system at 2-year follow-ups, the WOMAC and SF-36 systems showed a negative trend but no significant differences.

## Conclusion

Taken together, we found the post-operative knee skyline X-ray effective in evaluating the patella tilt. An increased patella tilt greater than 10° is associated with a worse score of specific components of knee society score at post-operative 2 years, regardless of the patella resurfacing status. Further study would focus on identifying components that lead to patella tilt and their effect on functional outcomes individually. Our result would help in prognosticating and post-operative counseling patients and help the surgeon identify the reason for excessive patella tilt and its relationship with the functional score in follow-up.

### Supplementary Information

Below is the link to the electronic supplementary material.

Supplementary file1 (DOCX 22 kb)

## Abbreviations

PT: Patellar tilt
PD: Patellar displacement
CPT: Combined patellar tilt
SD: Standard deviation
SF-36: Short Form-36
KSS: Knee Society Score
WOMAC: Western Ontario McMaster University Osteoarthritis Index
