# Supplementary material for: Post-operative Patellar Tilt More than 10° Can Affect Certain Components of Knee Society Score After Total Knee Arthroplasty at 2-Year Follow-Up
Source: Indian J Orthop. 2024 Feb 19;58(4):387–95. doi: 10.1007/s43465-023-01077-0 (PMC10963682; doi:10.1007/s43465-023-01077-0)
Supplement: Supplementary file 1 — Supplementary file1 (DOCX 22 kb) [file 43465_2023_1077_MOESM1_ESM.docx]

***Supplementary Tables:***

**Suppl Table 1: Comparison of radiological outcomes in non-resurfaced and resurfaced patella group**

|  | Non-resurfaced group  Mean ± SD | Resurfaced group  Mean ± SD |  |
| --- | --- | --- | --- |
|  | PT | CPT | Mean ± SE,  P value |
| Pre-op | 5.96±4.56 | 5.36 ± 4.26 | -0.60±0.58, p=0.302 |
| Post-op | 4.26±3.97 | 3.65±4.16 | 0.61 ±0.54, p=0.260 |
|  | PD | PD | Mean Diff± SD,  P value |
| Pre-op | 0.65±4.50 | 0.18±0.51 | -0.47±0.43,p=0.284 |
| Post-op | 0.11±0.32 | 0.07±0.18 | -0.05±0.03, p=0.176 |

Abbreviations: PT: Patellar tilt; PD: Patellar displacement; CPT: Combined patellar tilt; SD: Standard deviation; SE: Standard Error of the Mean; Diff: Differentiation

**Suppl Table 2: Comparison of functional outcomes in non-resurfaced and resurfaced patella group**

|  | Non-resurfaced group  Mean ± SD | Resurfaced group  Mean ± SD | Mean Diff± SE,  P value |
| --- | --- | --- | --- |
| PCS 0 | 31.52±7.21 | 31.97±6.77 | 0.45± 0.92, p=0.625 |
| MCS 0 | 57.65±6.70 | 56.14±8.22 | -1.50±0.98, p=0.128 |
| KSS-Knee 0 | 39.00±16.58 | 50.90±17.10 | -2.20±2.06, p=0.286 |
| KSS-Function 0 | 49.61±20.25 | 36.80±14.52 | 1.29± 2.48, p=0.605 |
| WOMAC 0 | 62.26±13.10 | 62.83±12.15 | 0.57± 1.67, p=0.733 |
| PCS 1 | 47.42± 7.19 | 47.89±7.21 | 0.48±0.95, p=0.616 |
| MCS 1 | 58.75±3.16 | 58.07± 5.30 | -0.68± 0.57, p=0.234 |
| KSS-Knee 1 | 92.26±12.51 | 76.32±17.83 | -0.15±1.71, p=0.930 |
| KSS-Function 1 | 74.63±17.49 | 92.41±12.88 | 1.69± 2.33, p=0.469 |
| WOMAC 1 | 88.00±8.97 | 88.69±8.87 | -0.69±1.18, p=0.558 |
| PCS 2 | 47.58±7.06 | 48.45±7.16 | 0.87 ±0.97, p=0.373 |
| MCS 2 | 59.03±4.16 | 58.97±3.59 | -0.05± 0.54, p=0.922 |
| KSS-Knee 2 | 92.95±11.76 | 78.38±16.16 | -1.04 ±1.61, p=0.519 |
| KSS-Function 2 | 75.30±17.33 | 93.99±9.28 | 3.08± 2.30, p=0.183 |
| WOMAC 2 | 88.82±7.09 | 89.30±6.72 | -0.48±0.95, p=0.612 |

All scores at preoperative and postoperative 1 year and 2 year are named by suffix. e.g. PCS 1 0 is the PCS score at preoperative evaluation; PCS 1 is the PCS score at postoperative 1 year follow-up, PCS 2 is the PCS score at postoperative 2 years follow-up.

Abbreviations: PCS: Physical component Score of Short Form-36; MCS: Mental Component Score of Short Form-36; KSS-Knee: The part one of Knee Society Score; KSS-Function: The part two of Knee Society Score; WOMAC: Western Ontario McMaster University Osteoarthritis Index.
